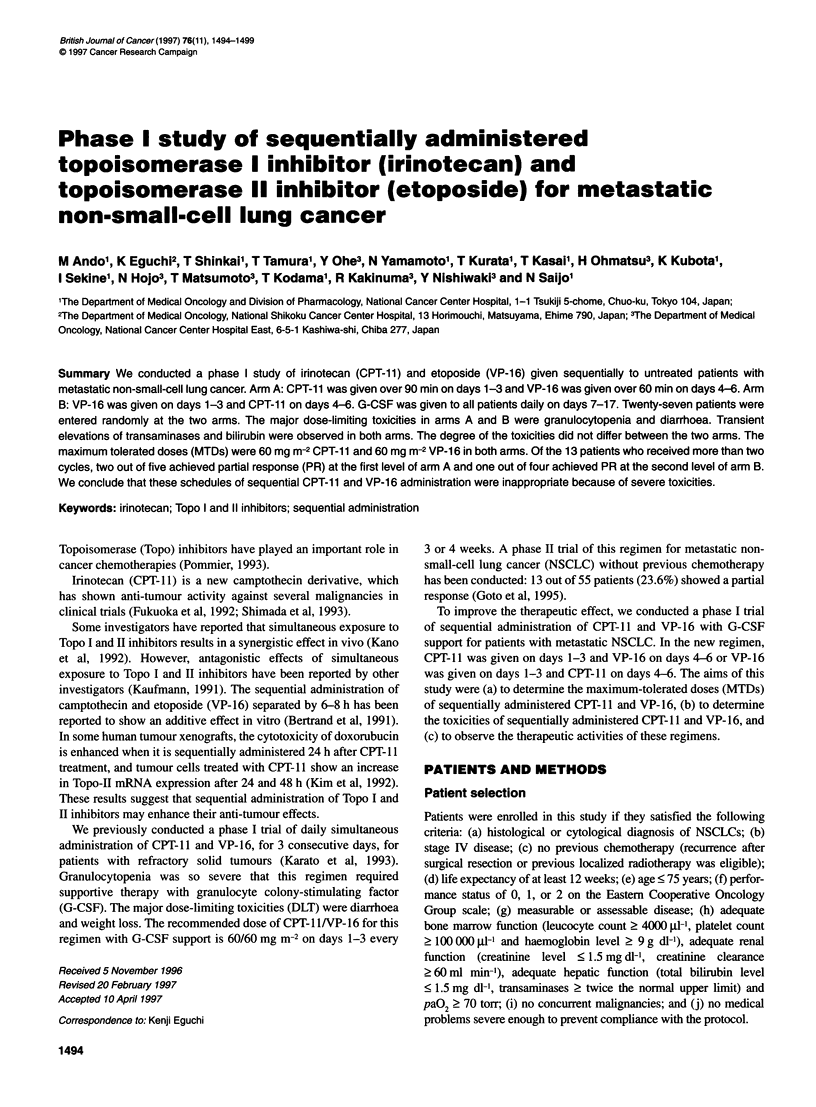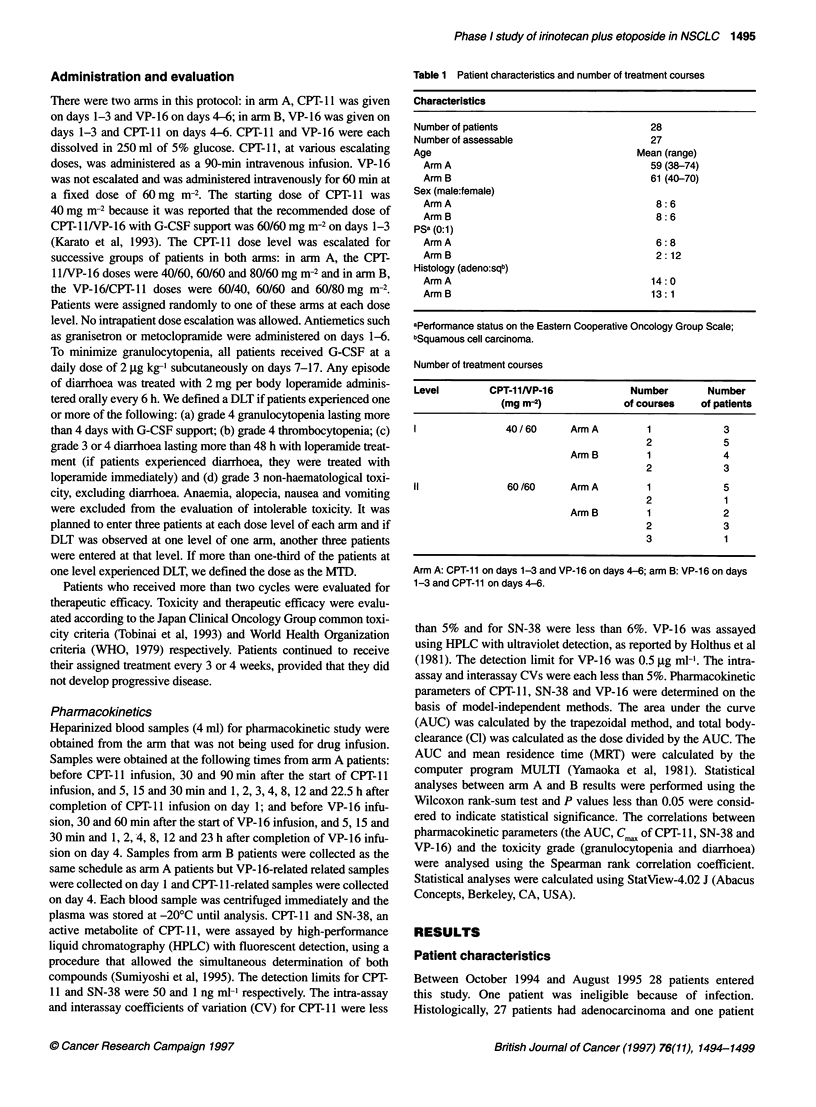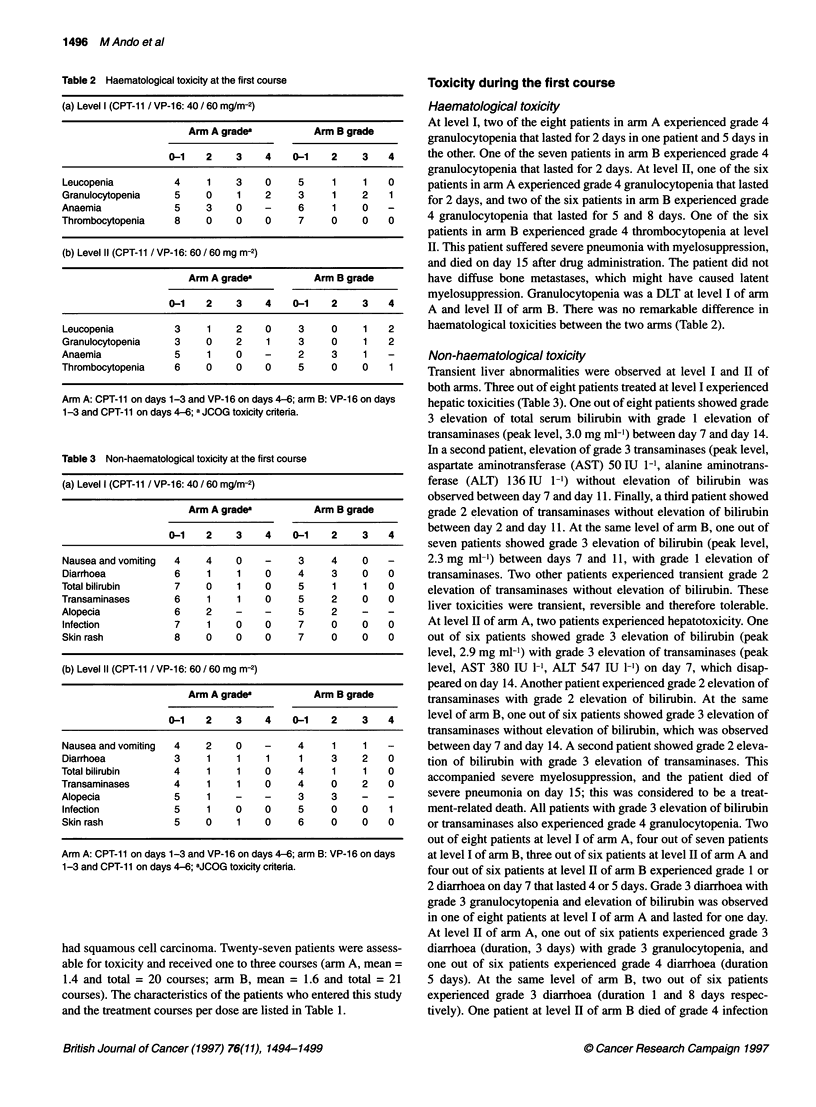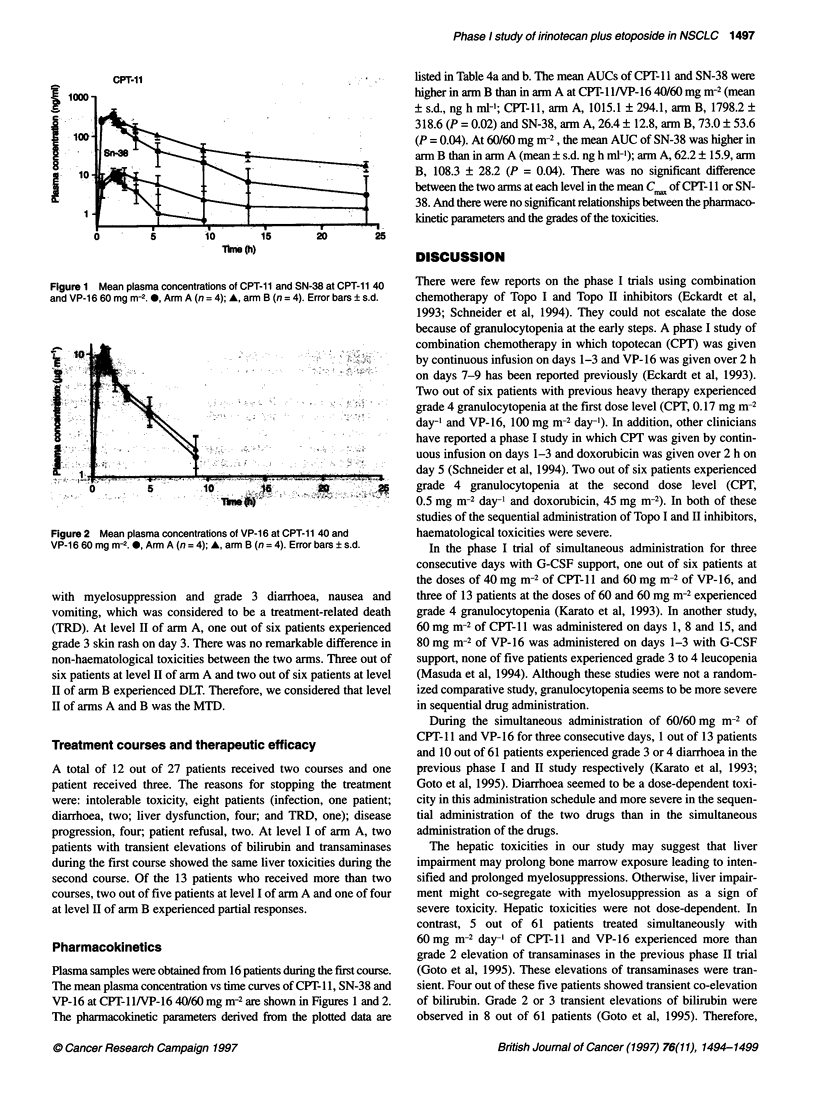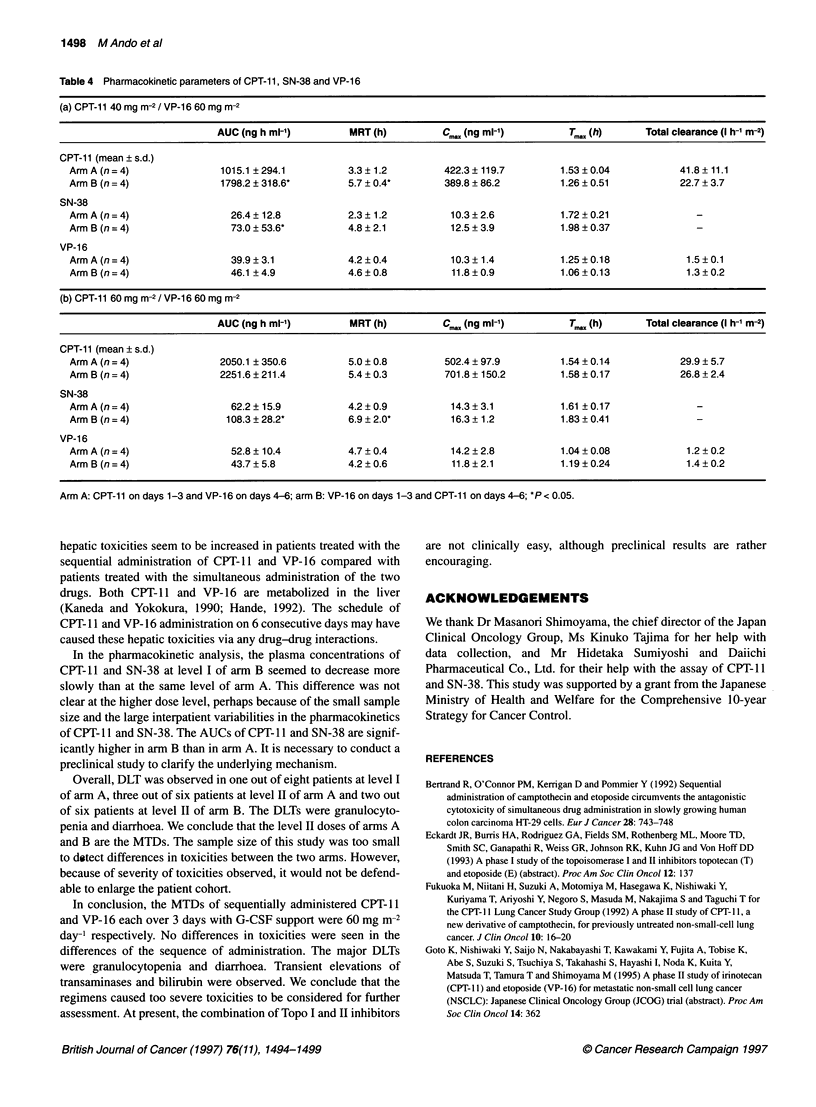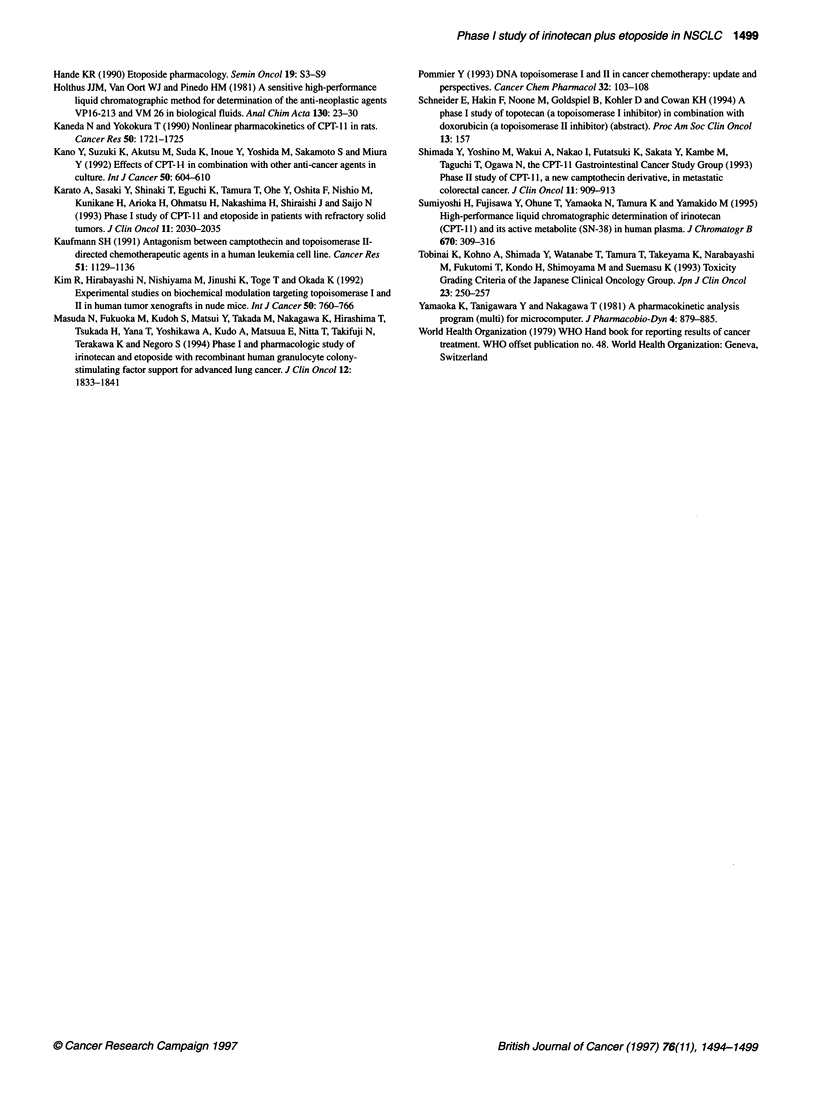# Phase I study of sequentially administered topoisomerase I inhibitor (irinotecan) and topoisomerase II inhibitor (etoposide) for metastatic non-small-cell lung cancer.

**DOI:** 10.1038/bjc.1997.584

**Published:** 1997

**Authors:** M. Ando, K. Eguchi, T. Shinkai, T. Tamura, Y. Ohe, N. Yamamoto, T. Kurata, T. Kasai, H. Ohmatsu, K. Kubota, I. Sekine, N. Hojo, T. Matsumoto, T. Kodama, R. Kakinuma, Y. Nishiwaki, N. Saijo

**Affiliations:** The Department of Medical Oncology, National Cancer Center Hospital, Tokyo, Japan.

## Abstract

**Images:**


British Journal of Cancer (1997) 76(11), 1494–1499
© 1997 Cancer Research Campaign

### Phase I study of sequentially administered topoisomerase I inhibitor (irinotecan) and topoisomerase II inhibitor (etoposide) for metastatic non-small-cell lung cancer

**M Ando[1], K Eguchi[2], T Shinkai[1], T Tamura[1], Y Ohe[3], N Yamamoto[1], T Kurata[1], T Kasai[1], H Ohmatsu[3], K Kubota[1], I Sekine[1], N Hojo[3], T Matsumoto[3], T Kodama[1], R Kakinuma[3], Y Nishiwaki[3] and N Saijo[1]**

[1]The Department of Medical Oncology and Division of Pharmacology, National Cancer Center Hospital, 1-1 Tsukiji 5-chome, Chuo-ku, Tokyo 104, Japan; [2]The Department of Medical Oncology, National Shikoku Cancer Center Hospital, 13 Horimouchi, Matsuyama, Ehime 790, Japan; [3]The Department of Medical Oncology, National Cancer Center Hospital East, 6-5-1 Kashiwa-shi, Chiba 277, Japan

**Summary** We conducted a phase I study of irinotecan (CPT-11) and etoposide (VP-16) given sequentially to untreated patients with metastatic non-small-cell lung cancer. Arm A: CPT-11 was given over 90 min on days 1–3 and VP-16 was given over 60 min on days 4–6. Arm B: VP-16 was given on days 1–3 and CPT-11 on days 4–6. G-CSF was given to all patients daily on days 7–17. Twenty-seven patients were entered randomly at the two arms. The major dose-limiting toxicities in arms A and B were granulocytopenia and diarrhoea. Transient elevations of transaminases and bilirubin were observed in both arms. The degree of the toxicities did not differ between the two arms. The maximum tolerated doses (MTDs) were 60 mg $m^{-2}$ CPT-11 and 60 mg $m^{-2}$ VP-16 in both arms. Of the 13 patients who received more than two cycles, two out of five achieved partial response (PR) at the first level of arm A and one out of four achieved PR at the second level of arm B. We conclude that these schedules of sequential CPT-11 and VP-16 administration were inappropriate because of severe toxicities.

**Keywords:** irinotecan; Topo I and II inhibitors; sequential administration

Topoisomerase (Topo) inhibitors have played an important role in cancer chemotherapies (Pommier, 1993).

Irinotecan (CPT-11) is a new camptothecin derivative, which has shown anti-tumour activity against several malignancies in clinical trials (Fukuoka et al, 1992; Shimada et al, 1993).

Some investigators have reported that simultaneous exposure to Topo I and II inhibitors results in a synergistic effect in vivo (Kano et al, 1992). However, antagonistic effects of simultaneous exposure to Topo I and II inhibitors have been reported by other investigators (Kaufmann, 1991). The sequential administration of camptothecin and etoposide (VP-16) separated by 6–8 h has been reported to show an additive effect in vitro (Bertrand et al, 1991). In some human tumour xenografts, the cytotoxicity of doxorubicin is enhanced when it is sequentially administered 24 h after CPT-11 treatment, and tumour cells treated with CPT-11 show an increase in Topo-II mRNA expression after 24 and 48 h (Kim et al, 1992). These results suggest that sequential administration of Topo I and II inhibitors may enhance their anti-tumour effects.

We previously conducted a phase I trial of daily simultaneous administration of CPT-11 and VP-16, for 3 consecutive days, for patients with refractory solid tumours (Karato et al, 1993). Granulocytopenia was so severe that this regimen required supportive therapy with granulocyte colony-stimulating factor (G-CSF). The major dose-limiting toxicities (DLT) were diarrhoea and weight loss. The recommended dose of CPT-11/VP-16 for this regimen with G-CSF support is 60/60 mg $m^{-2}$ on days 1–3 every 3 or 4 weeks. A phase II trial of this regimen for metastatic non-small-cell lung cancer (NSCLC) without previous chemotherapy has been conducted: 13 out of 55 patients (23.6%) showed a partial response (Goto et al, 1995).

*Received 5 November 1996*
*Revised 20 February 1997*
*Accepted 10 April 1997*

*Correspondence to:* Kenji Eguchi

To improve the therapeutic effect, we conducted a phase I trial of sequential administration of CPT-11 and VP-16 with G-CSF support for patients with metastatic NSCLC. In the new regimen, CPT-11 was given on days 1–3 and VP-16 on days 4–6 or VP-16 was given on days 1–3 and CPT-11 on days 4–6. The aims of this study were (a) to determine the maximum-tolerated doses (MTDs) of sequentially administered CPT-11 and VP-16, (b) to determine the toxicities of sequentially administered CPT-11 and VP-16, and (c) to observe the therapeutic activities of these regimens.

### PATIENTS AND METHODS

### Patient selection

Patients were enrolled in this study if they satisfied the following criteria: (a) histological or cytological diagnosis of NSCLCs; (b) stage IV disease; (c) no previous chemotherapy (recurrence after surgical resection or previous localized radiotherapy was eligible); (d) life expectancy of at least 12 weeks; (e) age $\leq$ 75 years; (f) performance status of 0, 1, or 2 on the Eastern Cooperative Oncology Group scale; (g) measurable or assessable disease; (h) adequate bone marrow function (leucocyte count $\geq$ 4000 $\mu l^{-1}$, platelet count $\geq$ 100 000 $\mu l^{-1}$ and haemoglobin level $\geq$ 9 g $dl^{-1}$), adequate renal function (creatinine level $\leq$ 1.5 mg $dl^{-1}$, creatinine clearance $\geq$ 60 ml $min^{-1}$), adequate hepatic function (total bilirubin level $\leq$ 1.5 mg $dl^{-1}$, transaminases $\geq$ twice the normal upper limit) and $paO_2 \geq$ 70 torr; (i) no concurrent malignancies; and (j) no medical problems severe enough to prevent compliance with the protocol.

### Administration and evaluation

There were two arms in this protocol: in arm A, CPT-11 was given on days 1–3 and VP-16 on days 4–6; in arm B, VP-16 was given on days 1–3 and CPT-11 on days 4–6. CPT-11 and VP-16 were each dissolved in 250 ml of 5% glucose. CPT-11, at various escalating doses, was administered as a 90-min intravenous infusion. VP-16 was not escalated and was administered intravenously for 60 min at a fixed dose of 60 mg $m^{-2}$. The starting dose of CPT-11 was 40 mg $m^{-2}$ because it was reported that the recommended dose of CPT-11/VP-16 with G-CSF support was 60/60 mg $m^{-2}$ on days 1–3 (Karato et al, 1993). The CPT-11 dose level was escalated for successive groups of patients in both arms: in arm A, the CPT-11/VP-16 doses were 40/60, 60/60 and 80/60 mg $m^{-2}$ and in arm B, the VP-16/CPT-11 doses were 60/40, 60/60 and 60/80 mg $m^{-2}$. Patients were assigned randomly to one of these arms at each dose level. No intrapatient dose escalation was allowed. Antiemetics such as granisetron or metoclopramide were administered on days 1–6. To minimize granulocytopenia, all patients received G-CSF at a daily dose of 2 μg $kg^{-1}$ subcutaneously on days 7–17. Any episode of diarrhoea was treated with 2 mg per body loperamide administered orally every 6 h. We defined a DLT if patients experienced one or more of the following: (a) grade 4 granulocytopenia lasting more than 4 days with G-CSF support; (b) grade 4 thrombocytopenia; (c) grade 3 or 4 diarrhoea lasting more than 48 h with loperamide treatment (if patients experienced diarrhoea, they were treated with loperamide immediately) and (d) grade 3 non-haematological toxicity, excluding diarrhoea. Anaemia, alopecia, nausea and vomiting were excluded from the evaluation of intolerable toxicity. It was planned to enter three patients at each dose level of each arm and if DLT was observed at one level of one arm, another three patients were entered at that level. If more than one-third of the patients at one level experienced DLT, we defined the dose as the MTD.

Patients who received more than two cycles were evaluated for therapeutic efficacy. Toxicity and therapeutic efficacy were evaluated according to the Japan Clinical Oncology Group common toxicity criteria (Tobinai et al, 1993) and World Health Organization criteria (WHO, 1979) respectively. Patients continued to receive their assigned treatment every 3 or 4 weeks, provided that they did not develop progressive disease.

### *Pharmacokinetics*

Heparinized blood samples (4 ml) for pharmacokinetic study were obtained from the arm that was not being used for drug infusion. Samples were obtained at the following times from arm A patients: before CPT-11 infusion, 30 and 90 min after the start of CPT-11 infusion, and 5, 15 and 30 min and 1, 2, 3, 4, 8, 12 and 22.5 h after completion of CPT-11 infusion on day 1; and before VP-16 infusion, 30 and 60 min after the start of VP-16 infusion, and 5, 15 and 30 min and 1, 2, 4, 8, 12 and 23 h after completion of VP-16 infusion on day 4. Samples from arm B patients were collected as the same schedule as arm A patients but VP-16-related related samples were collected on day 1 and CPT-11-related samples were collected on day 4. Each blood sample was centrifuged immediately and the plasma was stored at –20°C until analysis. CPT-11 and SN-38, an active metabolite of CPT-11, were assayed by high-performance liquid chromatography (HPLC) with fluorescent detection, using a procedure that allowed the simultaneous determination of both compounds (Sumiyoshi et al, 1995). The detection limits for CPT-11 and SN-38 were 50 and 1 ng $ml^{-1}$ respectively. The intra-assay and interassay coefficients of variation (CV) for CPT-11 were less than 5% and for SN-38 were less than 6%. VP-16 was assayed using HPLC with ultraviolet detection, as reported by Holthus et al (1981). The detection limit for VP-16 was 0.5 μg $ml^{-1}$. The intra-assay and interassay CVs were each less than 5%. Pharmacokinetic parameters of CPT-11, SN-38 and VP-16 were determined on the basis of model-independent methods. The area under the curve (AUC) was calculated by the trapezoidal method, and total body-clearance (Cl) was calculated as the dose divided by the AUC. The AUC and mean residence time (MRT) were calculated by the computer program MULTI (Yamaoka et al, 1981). Statistical analyses between arm A and B results were performed using the Wilcoxon rank-sum test and $P$ values less than 0.05 were considered to indicate statistical significance. The correlations between pharmacokinetic parameters (the AUC, $C_{max}$ of CPT-11, SN-38 and VP-16) and the toxicity grade (granulocytopenia and diarrhoea) were analysed using the Spearman rank correlation coefficient. Statistical analyses were calculated using StatView-4.02 J (Abacus Concepts, Berkeley, CA, USA).

**Table 1** Patient characteristics and number of treatment courses

| Characteristics | |
|---|---|
| Number of patients | 28 |
| Number of assessable | 27 |
| Age | Mean (range) |
| Arm A | 59 (38–74) |
| Arm B | 61 (40–70) |
| Sex (male:female) | |
| Arm A | 8 : 6 |
| Arm B | 8 : 6 |
| PS[a] (0:1) | |
| Arm A | 6 : 8 |
| Arm B | 2 : 12 |
| Histology (adeno:sq[b]) | |
| Arm A | 14 : 0 |
| Arm B | 13 : 1 |

[a]Performance status on the Eastern Cooperative Oncology Group Scale; [b]Squamous cell carcinoma.

Number of treatment courses

| Level | CPT-11/VP-16 (mg $m^{-2}$) | | Number of courses | Number of patients |
|---|---|---|---|---|
| I | 40 / 60 | Arm A | 1 | 3 |
| | | | 2 | 5 |
| | | Arm B | 1 | 4 |
| | | | 2 | 3 |
| II | 60 /60 | Arm A | 1 | 5 |
| | | | 2 | 1 |
| | | Arm B | 1 | 2 |
| | | | 2 | 3 |
| | | | 3 | 1 |

Arm A: CPT-11 on days 1–3 and VP-16 on days 4–6; arm B: VP-16 on days 1–3 and CPT-11 on days 4–6.

### RESULTS

### Patient characteristics

Between October 1994 and August 1995 28 patients entered this study. One patient was ineligible because of infection. Histologically, 27 patients had adenocarcinoma and one patient

© Cancer Research Campaign 1997

**Table 2** Haematological toxicity at the first course

(a) Level I (CPT-11 / VP-16: 40 / 60 mg/m-2)

| | Arm A grade[a] | | | | Arm B grade | | | |
|---|---|---|---|---|---|---|---|---|
| | 0–1 | 2 | 3 | 4 | 0–1 | 2 | 3 | 4 |
| Leucopenia | 4 | 1 | 3 | 0 | 5 | 1 | 1 | 0 |
| Granulocytopenia | 5 | 0 | 1 | 2 | 3 | 1 | 2 | 1 |
| Anaemia | 5 | 3 | 0 | – | 6 | 1 | 0 | – |
| Thrombocytopenia | 8 | 0 | 0 | 0 | 7 | 0 | 0 | 0 |

(b) Level II (CPT-11 / VP-16: 60 / 60 mg m-2)

| | Arm A grade[a] | | | | Arm B grade | | | |
|---|---|---|---|---|---|---|---|---|
| | 0–1 | 2 | 3 | 4 | 0–1 | 2 | 3 | 4 |
| Leucopenia | 3 | 1 | 2 | 0 | 3 | 0 | 1 | 2 |
| Granulocytopenia | 3 | 0 | 2 | 1 | 3 | 0 | 1 | 2 |
| Anaemia | 5 | 1 | 0 | – | 2 | 3 | 1 | – |
| Thrombocytopenia | 6 | 0 | 0 | 0 | 5 | 0 | 0 | 1 |

Arm A: CPT-11 on days 1–3 and VP-16 on days 4–6; arm B: VP-16 on days 1–3 and CPT-11 on days 4–6; [a] JCOG toxicity criteria.

**Table 3** Non-haematological toxicity at the first course

(a) Level I (CPT-11 / VP-16: 40 / 60 mg/m-2)

| | Arm A grade[a] | | | | Arm B grade | | | |
|---|---|---|---|---|---|---|---|---|
| | 0–1 | 2 | 3 | 4 | 0–1 | 2 | 3 | 4 |
| Nausea and vomiting | 4 | 4 | 0 | – | 3 | 4 | 0 | – |
| Diarrhoea | 6 | 1 | 1 | 0 | 4 | 3 | 0 | 0 |
| Total bilirubin | 7 | 0 | 1 | 0 | 5 | 1 | 1 | 0 |
| Transaminases | 6 | 1 | 1 | 0 | 5 | 2 | 0 | 0 |
| Alopecia | 6 | 2 | – | – | 5 | 2 | – | – |
| Infection | 7 | 1 | 0 | 0 | 7 | 0 | 0 | 0 |
| Skin rash | 8 | 0 | 0 | 0 | 7 | 0 | 0 | 0 |

(b) Level II (CPT-11 / VP-16: 60 / 60 mg m-2)

| | Arm A grade[a] | | | | Arm B grade | | | |
|---|---|---|---|---|---|---|---|---|
| | 0–1 | 2 | 3 | 4 | 0–1 | 2 | 3 | 4 |
| Nausea and vomiting | 4 | 2 | 0 | – | 4 | 1 | 1 | – |
| Diarrhoea | 3 | 1 | 1 | 1 | 1 | 3 | 2 | 0 |
| Total bilirubin | 4 | 1 | 1 | 0 | 4 | 1 | 1 | 0 |
| Transaminases | 4 | 1 | 1 | 0 | 4 | 0 | 2 | 0 |
| Alopecia | 5 | 1 | – | – | 3 | 3 | – | – |
| Infection | 5 | 1 | 0 | 0 | 5 | 0 | 0 | 1 |
| Skin rash | 5 | 0 | 1 | 0 | 6 | 0 | 0 | 0 |

Arm A: CPT-11 on days 1–3 and VP-16 on days 4–6; arm B: VP-16 on days 1–3 and CPT-11 on days 4–6; [a]JCOG toxicity criteria.

had squamous cell carcinoma. Twenty-seven patients were assessable for toxicity and received one to three courses (arm A, mean = 1.4 and total = 20 courses; arm B, mean = 1.6 and total = 21 courses). The characteristics of the patients who entered this study and the treatment courses per dose are listed in Table 1.

### Toxicity during the first course

### *Haematological toxicity*

At level I, two of the eight patients in arm A experienced grade 4 granulocytopenia that lasted for 2 days in one patient and 5 days in the other. One of the seven patients in arm B experienced grade 4 granulocytopenia that lasted for 2 days. At level II, one of the six patients in arm A experienced grade 4 granulocytopenia that lasted for 2 days, and two of the six patients in arm B experienced grade 4 granulocytopenia that lasted for 5 and 8 days. One of the six patients in arm B experienced grade 4 thrombocytopenia at level II. This patient suffered severe pneumonia with myelosuppression, and died on day 15 after drug administration. The patient did not have diffuse bone metastases, which might have caused latent myelosuppression. Granulocytopenia was a DLT at level I of arm A and level II of arm B. There was no remarkable difference in haematological toxicities between the two arms (Table 2).

### *Non-haematological toxicity*

Transient liver abnormalities were observed at level I and II of both arms. Three out of eight patients treated at level I experienced hepatic toxicities (Table 3). One out of eight patients showed grade 3 elevation of total serum bilirubin with grade 1 elevation of transaminases (peak level, 3.0 mg ml$^{-1}$) between day 7 and day 14. In a second patient, elevation of grade 3 transaminases (peak level, aspartate aminotransferase (AST) 50 IU l$^{-1}$, alanine aminotransferase (ALT) 136 IU l$^{-1}$) without elevation of bilirubin was observed between day 7 and day 11. Finally, a third patient showed grade 2 elevation of transaminases without elevation of bilirubin between day 2 and day 11. At the same level of arm B, one out of seven patients showed grade 3 elevation of bilirubin (peak level, 2.3 mg ml$^{-1}$) between days 7 and 11, with grade 1 elevation of transaminases. Two other patients experienced transient grade 2 elevation of transaminases without elevation of bilirubin. These liver toxicities were transient, reversible and therefore tolerable. At level II of arm A, two patients experienced hepatotoxicity. One out of six patients showed grade 3 elevation of bilirubin (peak level, 2.9 mg ml$^{-1}$) with grade 3 elevation of transaminases (peak level, AST 380 IU l$^{-1}$, ALT 547 IU l$^{-1}$) on day 7, which disappeared on day 14. Another patient experienced grade 2 elevation of transaminases with grade 2 elevation of bilirubin. At the same level of arm B, one out of six patients showed grade 3 elevation of transaminases without elevation of bilirubin, which was observed between day 7 and day 14. A second patient showed grade 2 elevation of bilirubin with grade 3 elevation of transaminases. This accompanied severe myelosuppression, and the patient died of severe pneumonia on day 15; this was considered to be a treatment-related death. All patients with grade 3 elevation of bilirubin or transaminases also experienced grade 4 granulocytopenia. Two out of eight patients at level I of arm A, four out of seven patients at level I of arm B, three out of six patients at level II of arm A and four out of six patients at level II of arm B experienced grade 1 or 2 diarrhoea on day 7 that lasted 4 or 5 days. Grade 3 diarrhoea with grade 3 granulocytopenia and elevation of bilirubin was observed in one of eight patients at level I of arm A and lasted for one day. At level II of arm A, one out of six patients experienced grade 3 diarrhoea (duration, 3 days) with grade 3 granulocytopenia, and one out of six patients experienced grade 4 diarrhoea (duration 5 days). At the same level of arm B, two out of six patients experienced grade 3 diarrhoea (duration 1 and 8 days respectively). One patient at level II of arm B died of grade 4 infection

© Cancer Research Campaign 1997

**Figure 1** Mean plasma concentrations of CPT-11 and SN-38 at CPT-11 40 and VP-16 60 mg $m^{-2}$. ●, Arm A ($n$ = 4); ▲, arm B ($n$ = 4). Error bars ± s.d.

**Figure 2** Mean plasma concentrations of VP-16 at CPT-11 40 and VP-16 60 mg $m^{-2}$. ●, Arm A ($n$ = 4); ▲, arm B ($n$ = 4). Error bars ± s.d.

with myelosuppression and grade 3 diarrhoea, nausea and vomiting, which was considered to be a treatment-related death (TRD). At level II of arm A, one out of six patients experienced grade 3 skin rash on day 3. There was no remarkable difference in non-haematological toxicities between the two arms. Three out of six patients at level II of arm A and two out of six patients at level II of arm B experienced DLT. Therefore, we considered that level II of arms A and B was the MTD.

### Treatment courses and therapeutic efficacy

A total of 12 out of 27 patients received two courses and one patient received three. The reasons for stopping the treatment were: intolerable toxicity, eight patients (infection, one patient; diarrhoea, two; liver dysfunction, four; and TRD, one); disease progression, four; patient refusal, two. At level I of arm A, two patients with transient elevations of bilirubin and transaminases during the first course showed the same liver toxicities during the second course. Of the 13 patients who received more than two courses, two out of five patients at level I of arm A and one of four at level II of arm B experienced partial responses.

### Pharmacokinetics

Plasma samples were obtained from 16 patients during the first course. The mean plasma concentration vs time curves of CPT-11, SN-38 and VP-16 at CPT-11/VP-16 40/60 mg $m^{-2}$ are shown in Figures 1 and 2. The pharmacokinetic parameters derived from the plotted data are listed in Table 4a and b. The mean AUCs of CPT-11 and SN-38 were higher in arm B than in arm A at CPT-11/VP-16 40/60 mg $m^{-2}$ (mean ± s.d., ng h $ml^{-1}$; CPT-11, arm A, 1015.1 ± 294.1, arm B, 1798.2 ± 318.6 ($P$ = 0.02) and SN-38, arm A, 26.4 ± 12.8, arm B, 73.0 ± 53.6 ($P$ = 0.04). At 60/60 mg $m^{-2}$, the mean AUC of SN-38 was higher in arm B than in arm A (mean ± s.d. ng h $ml^{-1}$); arm A, 62.2 ± 15.9, arm B, 108.3 ± 28.2 ($P$ = 0.04). There was no significant difference between the two arms at each level in the mean $C_{max}$ of CPT-11 or SN-38. And there were no significant relationships between the pharmacokinetic parameters and the grades of the toxicities.

### DISCUSSION

There were few reports on the phase I trials using combination chemotherapy of Topo I and Topo II inhibitors (Eckardt et al, 1993; Schneider et al, 1994). They could not escalate the dose because of granulocytopenia at the early steps. A phase I study of combination chemotherapy in which topotecan (CPT) was given by continuous infusion on days 1–3 and VP-16 was given over 2 h on days 7–9 has been reported previously (Eckardt et al, 1993). Two out of six patients with previous heavy therapy experienced grade 4 granulocytopenia at the first dose level (CPT, 0.17 mg $m^{-2}$ $day^{-1}$ and VP-16, 100 mg $m^{-2}$ $day^{-1}$). In addition, other clinicians have reported a phase I study in which CPT was given by continuous infusion on days 1–3 and doxorubicin was given over 2 h on day 5 (Schneider et al, 1994). Two out of six patients experienced grade 4 granulocytopenia at the second dose level (CPT, 0.5 mg $m^{-2}$ $day^{-1}$ and doxorubicin, 45 mg $m^{-2}$). In both of these studies of the sequential administration of Topo I and II inhibitors, haematological toxicities were severe.

In the phase I trial of simultaneous administration for three consecutive days with G-CSF support, one out of six patients at the doses of 40 mg $m^{-2}$ of CPT-11 and 60 mg $m^{-2}$ of VP-16, and three of 13 patients at the doses of 60 and 60 mg $m^{-2}$ experienced grade 4 granulocytopenia (Karato et al, 1993). In another study, 60 mg $m^{-2}$ of CPT-11 was administered on days 1, 8 and 15, and 80 mg $m^{-2}$ of VP-16 was administered on days 1–3 with G-CSF support, none of five patients experienced grade 3 to 4 leucopenia (Masuda et al, 1994). Although these studies were not a randomized comparative study, granulocytopenia seems to be more severe in sequential drug administration.

During the simultaneous administration of 60/60 mg $m^{-2}$ of CPT-11 and VP-16 for three consecutive days, 1 out of 13 patients and 10 out of 61 patients experienced grade 3 or 4 diarrhoea in the previous phase I and II study respectively (Karato et al, 1993; Goto et al, 1995). Diarrhoea seemed to be a dose-dependent toxicity in this administration schedule and more severe in the sequential administration of the two drugs than in the simultaneous administration of the drugs.

The hepatic toxicities in our study may suggest that liver impairment may prolong bone marrow exposure leading to intensified and prolonged myelosuppressions. Otherwise, liver impairment might co-segregate with myelosuppression as a sign of severe toxicity. Hepatic toxicities were not dose-dependent. In contrast, 5 out of 61 patients treated simultaneously with 60 mg $m^{-2}$ $day^{-1}$ of CPT-11 and VP-16 experienced more than grade 2 elevation of transaminases in the previous phase II trial (Goto et al, 1995). These elevations of transaminases were transient. Four out of these five patients showed transient co-elevation of bilirubin. Grade 2 or 3 transient elevations of bilirubin were observed in 8 out of 61 patients (Goto et al, 1995). Therefore,

© Cancer Research Campaign 1997

**Table 4** Pharmacokinetic parameters of CPT-11, SN-38 and VP-16

(a) CPT-11 40 mg $m^{-2}$ / VP-16 60 mg $m^{-2}$

| | AUC (ng h $ml^{-1}$) | MRT (h) | $C_{max}$ (ng $ml^{-1}$) | $T_{max}$ (*h*) | Total clearance (l $h^{-1}$ $m^{-2}$) |
|---|---|---|---|---|---|
| CPT-11 (mean ± s.d.) | | | | | |
| Arm A (*n* = 4) | 1015.1 ± 294.1 | 3.3 ± 1.2 | 422.3 ± 119.7 | 1.53 ± 0.04 | 41.8 ± 11.1 |
| Arm B (*n* = 4) | 1798.2 ± 318.6* | 5.7 ± 0.4* | 389.8 ± 86.2 | 1.26 ± 0.51 | 22.7 ± 3.7 |
| SN-38 | | | | | |
| Arm A (*n* = 4) | 26.4 ± 12.8 | 2.3 ± 1.2 | 10.3 ± 2.6 | 1.72 ± 0.21 | – |
| Arm B (*n* = 4) | 73.0 ± 53.6* | 4.8 ± 2.1 | 12.5 ± 3.9 | 1.98 ± 0.37 | – |
| VP-16 | | | | | |
| Arm A (*n* = 4) | 39.9 ± 3.1 | 4.2 ± 0.4 | 10.3 ± 1.4 | 1.25 ± 0.18 | 1.5 ± 0.1 |
| Arm B (*n* = 4) | 46.1 ± 4.9 | 4.6 ± 0.8 | 11.8 ± 0.9 | 1.06 ± 0.13 | 1.3 ± 0.2 |

(b) CPT-11 60 mg $m^{-2}$ / VP-16 60 mg $m^{-2}$

| | AUC (ng h $ml^{-1}$) | MRT (h) | $C_{max}$ (ng $ml^{-1}$) | $T_{max}$ (h) | Total clearance (l $h^{-1}$ $m^{-2}$) |
|---|---|---|---|---|---|
| CPT-11 (mean ± s.d.) | | | | | |
| Arm A (*n* = 4) | 2050.1 ± 350.6 | 5.0 ± 0.8 | 502.4 ± 97.9 | 1.54 ± 0.14 | 29.9 ± 5.7 |
| Arm B (*n* = 4) | 2251.6 ± 211.4 | 5.4 ± 0.3 | 701.8 ± 150.2 | 1.58 ± 0.17 | 26.8 ± 2.4 |
| SN-38 | | | | | |
| Arm A (*n* = 4) | 62.2 ± 15.9 | 4.2 ± 0.9 | 14.3 ± 3.1 | 1.61 ± 0.17 | – |
| Arm B (*n* = 4) | 108.3 ± 28.2* | 6.9 ± 2.0* | 16.3 ± 1.2 | 1.83 ± 0.41 | – |
| VP-16 | | | | | |
| Arm A (*n* = 4) | 52.8 ± 10.4 | 4.7 ± 0.4 | 14.2 ± 2.8 | 1.04 ± 0.08 | 1.2 ± 0.2 |
| Arm B (*n* = 4) | 43.7 ± 5.8 | 4.2 ± 0.6 | 11.8 ± 2.1 | 1.19 ± 0.24 | 1.4 ± 0.2 |

Arm A: CPT-11 on days 1–3 and VP-16 on days 4–6; arm B: VP-16 on days 1–3 and CPT-11 on days 4–6; $^{*}P < 0.05$.

hepatic toxicities seem to be increased in patients treated with the sequential administration of CPT-11 and VP-16 compared with patients treated with the simultaneous administration of the two drugs. Both CPT-11 and VP-16 are metabolized in the liver (Kaneda and Yokokura, 1990; Hande, 1992). The schedule of CPT-11 and VP-16 administration on 6 consecutive days may have caused these hepatic toxicities via any drug–drug interactions.

In the pharmacokinetic analysis, the plasma concentrations of CPT-11 and SN-38 at level I of arm B seemed to decrease more slowly than at the same level of arm A. This difference was not clear at the higher dose level, perhaps because of the small sample size and the large interpatient variabilities in the pharmacokinetics of CPT-11 and SN-38. The AUCs of CPT-11 and SN-38 are significantly higher in arm B than in arm A. It is necessary to conduct a preclinical study to clarify the underlying mechanism.

Overall, DLT was observed in one out of eight patients at level I of arm A, three out of six patients at level II of arm A and two out of six patients at level II of arm B. The DLTs were granulocytopenia and diarrhoea. We conclude that the level II doses of arms A and B are the MTDs. The sample size of this study was too small to detect differences in toxicities between the two arms. However, because of severity of toxicities observed, it would not be defendable to enlarge the patient cohort.

In conclusion, the MTDs of sequentially administered CPT-11 and VP-16 each over 3 days with G-CSF support were 60 mg $m^{-2}$ $day^{-1}$ respectively. No differences in toxicities were seen in the differences of the sequence of administration. The major DLTs were granulocytopenia and diarrhoea. Transient elevations of transaminases and bilirubin were observed. We conclude that the regimens caused too severe toxicities to be considered for further assessment. At present, the combination of Topo I and II inhibitors are not clinically easy, although preclinical results are rather encouraging.

### ACKNOWLEDGEMENTS

We thank Dr Masanori Shimoyama, the chief director of the Japan Clinical Oncology Group, Ms Kinuko Tajima for her help with data collection, and Mr Hidetaka Sumiyoshi and Daiichi Pharmaceutical Co., Ltd. for their help with the assay of CPT-11 and SN-38. This study was supported by a grant from the Japanese Ministry of Health and Welfare for the Comprehensive 10-year Strategy for Cancer Control.

### REFERENCES

Bertrand R, O'Connor PM, Kerrigan D and Pommier Y (1992) Sequential administration of camptothecin and etoposide circumvents the antagonistic cytotoxicity of simultaneous drug administration in slowly growing human colon carcinoma HT-29 cells. *Eur J Cancer* **28**: 743–748

Eckardt JR, Burris HA, Rodriguez GA, Fields SM, Rothenberg ML, Moore TD, Smith SC, Ganapathi R, Weiss GR, Johnson RK, Kuhn JG and Von Hoff DD (1993) A phase I study of the topoisomerase I and II inhibitors topotecan (T) and etoposide (E) (abstract). *Proc Am Soc Clin Oncol* **12**: 137

Fukuoka M, Niitani H, Suzuki A, Motomiya M, Hasegawa K, Nishiwaki Y, Kuriyama T, Ariyoshi Y, Negoro S, Masuda M, Nakajima S and Taguchi T for the CPT-11 Lung Cancer Study Group (1992) A phase II study of CPT-11, a new derivative of camptothecin, for previously untreated non-small-cell lung cancer. *J Clin Oncol* **10**: 16–20

Goto K, Nishiwaki Y, Saijo N, Nakabayashi T, Kawakami Y, Fujita A, Tobise K, Abe S, Suzuki S, Tsuchiya S, Takahashi S, Hayashi I, Noda K, Kuita Y, Matsuda T, Tamura T and Shimoyama M (1995) A phase II study of irinotecan (CPT-11) and etoposide (VP-16) for metastatic non-small cell lung cancer (NSCLC): Japanese Clinical Oncology Group (JCOG) trial (abstract). *Proc Am Soc Clin Oncol* **14**: 362

 *© Cancer Research Campaign 1997*

Hande KR (1990) Etoposide pharmacology. *Semin Oncol* **19**: S3–S9

Holthus JJM, Van Oort WJ and Pinedo HM (1981) A sensitive high-performance liquid chromatographic method for determination of the anti-neoplastic agents VP16-213 and VM 26 in biological fluids. *Anal Chim Acta* **130**: 23–30

Kaneda N and Yokokura T (1990) Nonlinear pharmacokinetics of CPT-11 in rats. *Cancer Res* **50**: 1721–1725

Kano Y, Suzuki K, Akutsu M, Suda K, Inoue Y, Yoshida M, Sakamoto S and Miura Y (1992) Effects of CPT-11 in combination with other anti-cancer agents in culture. *Int J Cancer* **50**: 604–610

Karato A, Sasaki Y, Shinaki T, Eguchi K, Tamura T, Ohe Y, Oshita F, Nishio M, Kunikane H, Arioka H, Ohmatsu H, Nakashima H, Shiraishi J and Saijo N (1993) Phase I study of CPT-11 and etoposide in patients with refractory solid tumors. *J Clin Oncol* **11**: 2030–2035

Kaufmann SH (1991) Antagonism between camptothecin and topoisomerase II-directed chemotherapeutic agents in a human leukemia cell line. *Cancer Res* **51**: 1129–1136

Kim R, Hirabayashi N, Nishiyama M, Jinushi K, Toge T and Okada K (1992) Experimental studies on biochemical modulation targeting topoisomerase I and II in human tumor xenografts in nude mice. *Int J Cancer* **50**: 760–766

Masuda N, Fukuoka M, Kudoh S, Matsui Y, Takada M, Nakagawa K, Hirashima T, Tsukada H, Yana T, Yoshikawa A, Kudo A, Matsuua E, Nitta T, Takifuji N, Terakawa K and Negoro S (1994) Phase I and pharmacologic study of irinotecan and etoposide with recombinant human granulocyte colony-stimulating factor support for advanced lung cancer. *J Clin Oncol* **12**: 1833–1841

Pommier Y (1993) DNA topoisomerase I and II in cancer chemotherapy: update and perspectives. *Cancer Chem Pharmacol* **32**: 103–108

Schneider E, Hakin F, Noone M, Goldspiel B, Kohler D and Cowan KH (1994) A phase I study of topotecan (a topoisomerase I inhibitor) in combination with doxorubicin (a topoisomerase II inhibitor) (abstract). *Proc Am Soc Clin Oncol* **13**: 157

Shimada Y, Yoshino M, Wakui A, Nakao I, Futatsuki K, Sakata Y, Kambe M, Taguchi T, Ogawa N, the CPT-11 Gastrointestinal Cancer Study Group (1993) Phase II study of CPT-11, a new camptothecin derivative, in metastatic colorectal cancer. *J Clin Oncol* **11**: 909–913

Sumiyoshi H, Fujisawa Y, Ohune T, Yamaoka N, Tamura K and Yamakido M (1995) High-performance liquid chromatographic determination of irinotecan (CPT-11) and its active metabolite (SN-38) in human plasma. *J Chromatogr B* **670**: 309–316

Tobinai K, Kohno A, Shimada Y, Watanabe T, Tamura T, Takeyama K, Narabayashi M, Fukutomi T, Kondo H, Shimoyama M and Suemasu K (1993) Toxicity Grading Criteria of the Japanese Clinical Oncology Group. *Jpn J Clin Oncol* **23**: 250–257

Yamaoka K, Tanigawara Y and Nakagawa T (1981) A pharmacokinetic analysis program (multi) for microcomputer. *J Pharmacobio-Dyn* **4**: 879–885.

World Health Organization (1979) WHO Hand book for reporting results of cancer treatment. WHO offset publication no. 48. World Health Organization: Geneva, Switzerland

© Cancer Research Campaign 1997